# Co-Occurrence of Major Mycotoxins and Emerging *Alternaria* Toxins in Couscous Marketed in Algeria

**DOI:** 10.3390/toxins17100483

**Published:** 2025-09-26

**Authors:** Sarah Mohammedi-Ameur, Terenzio Bertuzzi, Roberta Battaglia, Federico Siboni, Paola Giorni, Dahmane Mohammedi

**Affiliations:** 1 École Supérieure des Sciences de l’Aliment et des Industries Agroalimentaires (ESSAIA, Algiers), Oued Smar, Algiers 16200, Algeria; 2 Laboratoire de Recherche Santé et Productions Animales, École Nationale Supérieure Vétérinaire Rabie Bouchama (ENSV, Algiers), Bab Ezzouar, Algiers 16042, Algeria; 3 Department of Animal Science, Food and Nutrition (DIANA), Faculty of Agriculture, Food and Environmental Sciences, Università Cattolica del Sacro Cuore, 29122 Piacenza, Italy; 4 Department of Sustainable Crop Production (DI.PRO.VE.S.), Faculty of Agriculture, Food and Environmental Sciences, Università Cattolica del Sacro Cuore, 29122 Piacenza, Italy; paola.giorni@unicatt.it

**Keywords:** mycotoxins, co-occurrence, couscous, *Alternaria* toxins, Algeria

## Abstract

Cereal contamination by mycotoxins represents a major food safety concern. This study aimed to assess the co-occurrence of 15 mycotoxins in 50 couscous samples marketed in Algeria using HPLC/FLD and LC-MS/MS techniques. The samples included various couscous types, differing in ingredients, production method (organic or conventional), processing operations, and granularity. The most frequently detected mycotoxins were tentoxin (76%), deoxynivalenol (74%), tenuazonic acid (72%), and ochratoxin A (54%). For the regulated mycotoxins, none of the concentrations exceeded the maximum levels set by the European Union. In contrast, tenuazonic acid and tentoxin, which are not yet regulated, were the most common compounds detected. Contamination with multiple mycotoxins was commonly observed: 90% of the samples contained at least two mycotoxins, with some containing up to seven. The most frequent combination involved tenuazonic acid-tentoxin-ochratoxin A. These findings highlight the need for frequent and systematic monitoring of couscous and other processed cereal-based products.

## 1. Introduction

Cereal commodities can be contaminated with mycotoxins, which are considered among the most widespread contaminants in foods [1]. They are produced mainly by the Genera *Penicillium*, *Aspergillus*, *Fusarium*, *Alternaria* and *Claviceps* and their production can occur both in field and during storage and can vary depending on meteorological, agronomic and storage conditions. Mycotoxins mainly considered for their relevant toxicity on human and animal health include aflatoxins (AFs), ochratoxin A (OTA), trichothecenes (TCTs), zearalenone (ZEA), fumonisins (FBs) and *Alternaria* toxins (ALTs). Cereal-based products are staple foods in the Mediterranean area; however, in the past decade, few studies have been published on mycotoxin contamination in cereal products from Northern African countries [2,3,4,5,6,7,8]. Regarding mycotoxin contamination studies carried out in Algeria, widespread occurrence, sometimes at high levels, was found. Mahdjoubi et al. [5] reported that 65% of samples were contaminated with at least one toxin, while Belasli et al. [2] found incidences of 47.9% and 85.9% for AFs and DON, respectively, in cereal products. Algeria, which is bordered to the north by Mediterranean Sea, is a country usually characterized by hot and dry conditions in summer, but with some humid areas; these meteorological conditions can favor the growth of some mycotoxigenic fungi and the production of their related mycotoxins [9]. Khouni et al. [10] reported that several mycotoxins were detected in wheat samples collected at harvest and at different stages of storage in several regions of Algeria. Their findings showed widespread contamination with DON in samples from the Mediterranean zone, characterized by humid conditions, and a high frequency of OTA in the samples from regions with a hot summer climate. Despite the relevant contamination incidences found in these studies, in Algeria, regulatory limits currently apply only to aflatoxin B1 (10 µg/kg) and to the sum of aflatoxins (20 µg/kg) in peanuts, tree nuts, and cereals, while no national maximum levels have been defined for other foodstuffs [11]. The absence of continuous controls based on legal limits does not favor the development of preventive agricultural and storage practices. Moreover, in Algeria, many cereals are imported, prompting the need for stringent controls. The 2023/24 cereal import requirements (July 2023/June 2024) were forecasted at about 14 million tonnes, representing the highest volume of cereal imports compared to the previous five years. Wheat imports represent about 60% of total cereals imports and are forecast at 8 million tonnes [12]. On the other hand, no reliable data on the export of Algerian cereal-based products was found; the export of cereal products is subject to governmental restrictions since products manufactured from state-subsidized raw wheat are not allowed to be exported to prevent shortages in the local market and to safeguard national food security. It is also important to note that extended shipping routes can present a risk for the proliferation of toxigenic mold species, particularly under conditions of insufficient post-discharge inspection and control [6].

Couscous is a staple food in North Africa and the national dish in several countries such as Algeria, Tunisia, Libya and Morocco. Its origins lie deep within Berber culture [13], with the earliest traces of its traditional preparation tools, a form of couscous steamer, discovered in the Kabylie region of Algeria [14]. In Algeria, couscous exhibits significant diversity; numerous variations exist, corresponding to differences in regions, seasonal cycles, and social occasions [15]. Couscous is generally prepared using durum wheat, but it can also be made using other cereals, such as soft wheat, barley, corn and rice. Couscous can be homemade or industrially manufactured [16]; regarding market value, the Algerian couscous sector was estimated at USD 454.3 million in 2024. It is projected to grow at a compound annual growth rate of 5.14%, reaching approximately USD 730.2 million by 2033 [17]. Although couscous is a widely consumed staple food, its contamination by mycotoxins has received very limited investigation in the scientific literature [2,8]. In this context, it is essential to document the current state of knowledge regarding of mycotoxins in couscous produced and consumed in Algeria. This study represents one of the first contributions to the evaluation of mycotoxin co-occurrence in this traditional food product. The main objective was to perform a preliminary investigation of the presence of major and emerging mycotoxins, including aflatoxins (AFs), ochratoxin A (OTA), deoxynivalenol (DON), nivalenol (NIV), HT-2 and T-2 toxins, fumonisins (FBs), and *Alternaria* toxins (ALTs), in couscous samples collected across Algeria. Then, a small survey (50 samples) was carried out, collecting different types of couscous, based on the type of cereal used (wheat, barley, maize, rice), processing method (homemade or industrial), production system (organic or conventional), and granule size (large, medium, or fine). For each mycotoxin group, specific extraction, purification, and instrumental (LC-MS/MS or HPLC-FLD) methods were applied to achieve accurate quantitative results.

## 2. Results

**Figure 1:**
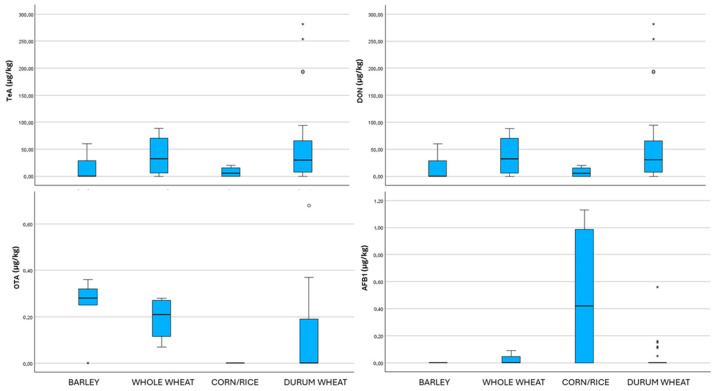
Box plot analysis of tenuazonic acid (TeA), deoxynivalenol (DON), ochratoxin A (OTA) and aflatoxin B1 (AFB1) concentrations (µg/kg) in the different couscous samples based on different cereals. Within each box, horizontal black lines indicate median values, boxes extend from the 25th to the 75th percentile of each group’s distribution of values, the lower and upper lines denote error lines, dots denote observations outside the 10th and 90th percentiles and asterisks denote extreme outliers.

Couscous is generally a durum wheat semolina-based product, but it can also be prepared using whole wheat, barley, or maize (sometimes including a low percentage of rice) and using different manufacturing processes, i.e., industrial or hand-rolled [16]. The co-occurrence of mycotoxins in the different types of couscous samples analyzed, as well as the total number of samples tested, mean concentrations, and maximum levels detected for each mycotoxin, are presented in Table 1, Table 2, Table 3, Table 4, Table 5 and Table 6 and box plots (Figure 1).

### 2.1. Occurrence of AFs in Couscous Samples

Among the hand-rolled wheat couscous samples analyzed (*n* = 11), aflatoxin B_1_ (AFB_1_) was detected in two cases: one sample containing basil, with a concentration of 0.11 µg/kg, and another composed solely of wheat, with a concentration of 0.56 µg/kg. In the latter sample, aflatoxin B_2_ (AFB_2_) was also detected at a level of 0.10 µg/kg. The mean concentrations of AFB_1_ and AFB_2_, using a value of LOD/2 for left-censored results, were 0.08 ± 0.16 µg/kg and 0.03 ± 0.02 µg/kg, respectively (Table 1).

Among the industrial wheat couscous samples (*n* = 23), four (17.4%) were found to be contaminated with AFB_1_ (Table 2), with very low concentrations ranging from 0.05 to 0.16 µg/kg. The mean AFB_1_ level was 0.041 ± 0.040 µg/kg. In contrast, no aflatoxin contamination was detected in the organic wheat couscous samples (*n* = 3) (Table 3), suggesting that larger sampling should be carried out to confirm this absence of contamination in organically produced couscous.

Among the corn-based couscous samples analyzed (*n* = 4), 50% were contaminated with AFB_1_, AFB_2_, and AFG_1_. The detected values ranged from 0.83 to 1.13 µg/kg for AFB_1_, from 0.05 to 0.09 µg/kg for AFB_2_, and from 0.40 to 0.45 µg/kg for AFG_1_. The corresponding mean concentrations were 0.51 µg/kg, 0.05 µg/kg, and 0.22 µg/kg, respectively. AFG_2_ was identified in a single sample at a concentration of 0.05 µg/kg (Table 4).

In contrast, no aflatoxin contamination was detected in any of the barley couscous samples (*n* = 5) (Table 5) or in those made from whole wheat (*n* = 4) (Table 6).

### 2.2. Occurrence of Fusarium Mycotoxins in Couscous Samples

No contamination with fumonisin FB_1_ or FB_2_ was detected in the hand-rolled, industrial, organic, barley, or whole wheat couscous samples (Table 1, Table 2, Table 3, Table 5 and Table 6). By comparison, all corn-based couscous samples were contaminated with FB_1_, with concentrations ranging from 37.5 to 659.9 µg/kg and a mean level of 309.8 µg/kg. FB_2_ was likewise present in all these samples, with concentrations between 8.7 and 249.9 µg/kg, and an average value of 120.6 µg/kg (Table 4).

Regarding DON contamination, the highest levels were observed in organic wheat couscous samples, with a maximum concentration of 281.5 µg/kg; the mean value for this category was 200.3 µg/kg.

In addition, seven hand-rolled wheat couscous samples (63.6%) were contaminated with DON at levels ranging from 22.4 to 194.1 µg/kg, with an average concentration of 47.7 µg/kg.

DON was also detected in 20 industrial wheat couscous samples (86.9%), with concentrations between 5 and 192.4 µg/kg.

Whole wheat couscous and barley couscous samples were also found to be contaminated with DON, with levels ranging from 12.2 to 88.6 µg/kg for whole wheat and from 28.6 to 60.1 µg/kg for barley-based couscous. The mean contamination levels were 38.9 and 19.2 µg/kg for whole wheat and barley couscous, respectively. Low incidence (50%) and concentration levels were found in corn couscous samples.

Regarding NIV, no contamination was detected in any of the couscous samples analyzed, regardless of their type or origin.

According to the analytical results, HT-2 toxin was not detected in any of the corn couscous samples. In contrast, it was detected in a single hand-rolled wheat couscous sample, with a concentration of 8.75 µg/kg.

Among the industrial wheat couscous samples, six samples (26.1%) were contaminated with HT-2, at concentrations ranging from 0.30 to 1.84 µg/kg.

For barley couscous, four samples (80%) tested positive for HT-2, with concentrations ranging from 1.44 to 8.44 µg/kg; the mean concentration calculated for all samples was 4.36 µg/kg.

HT-2 was also detected in two whole wheat couscous samples (50%), with levels ranging from 1.29 to 1.93 µg/kg and an overall mean of 0.83 µg/kg.

No contamination with T-2 toxin was detected in any of the corn, whole wheat couscous, or organic wheat couscous samples. In contrast, one hand-rolled wheat couscous sample (9.1%) was found to contain T-2 at a concentration of 2.76 µg/kg.

Among the industrial wheat couscous samples, four samples (17.4%) tested positive for T-2 toxin, with concentrations ranging from 0.10 to 0.80 µg/kg. For barley couscous, four samples (80%) were contaminated with T-2, with levels ranging from 0.58 to 2.77 µg/kg, and an overall mean value of 1.03 µg/kg, showing a higher incidence.

### 2.3. Occurrence of OTA in Couscous Samples

Regarding the presence of ochratoxin A (OTA) in the analyzed samples, six hand-rolled wheat couscous samples (54.5%) were found to be contaminated, with low concentrations ranging from 0.06 to 0.68 µg/kg, and a mean value of 0.07 µg/kg.

Among the industrial wheat couscous samples, nine samples (39.13%) tested positive for OTA, with levels ranging from 0.04 to 0.37 µg/kg. All the organic wheat couscous and whole wheat couscous samples were contaminated with OTA. Concentrations ranged from 0.10 to 0.36 µg/kg (mean: 0.22 µg/kg) for the organic wheat samples, and from 0.07 to 0.28 µg/kg (mean: 0.19 µg/kg) for the whole wheat samples. For the barley-based couscous, four samples (80%) were contaminated, with OTA levels between 0.25 and 0.36 µg/kg, and an overall mean value of 0.24 µg/kg. In contrast to all other sample types, no OTA contamination was detected in the corn-based couscous samples.

Differences in OTA contamination were found between the different ingredients used for couscous preparation; in particular, couscous that contained barley was more contaminated than the corn, whole wheat and wheat couscous.

### 2.4. Occurrence of Emerging Mycotoxins in Couscous Samples

No contamination with alternariol (AOH) or alternariol monomethyl ether (AME) was detected in any of the couscous samples analyzed. In contrast, tenuazonic acid (TeA) was frequently found. It was detected in 81.8% of hand-rolled wheat couscous samples, with concentrations ranging from 24.8 to 162.7 µg/kg and a mean level of 71.8 µg/kg. Among the industrial wheat couscous samples, 19 (82.6%) were contaminated with TeA, with levels ranging from 15.8 to 117.4 µg/kg and an average of 39.1 µg/kg. All organic wheat couscous samples (*n* = 3) were contaminated, with TeA levels ranging from 78.9 to 141.5 µg/kg and a mean of 117.5 µg/kg. Only one barley-based couscous sample (20%) was contaminated, with a concentration of 19.3 µg/kg and an average of 5.9 µg/kg. All whole wheat couscous samples (*n* = 4) contained TeA, with concentrations between 41.2 and 54.3 µg/kg and an average level of 47.8 µg/kg. In contrast, no TeA contamination was detected in any of the corn-based couscous samples.

All hand-rolled wheat couscous and whole wheat couscous samples (100%) were found to be contaminated with tentoxin (TEN), with concentrations ranging from 1.1 to 4.8 µg/kg (mean: 2.9 µg/kg) and from 2.3 to 6.6 µg/kg (mean: 4.1 µg/kg), respectively. Among the industrial wheat couscous samples, 17 (73.9%) were contaminated with TEN, with levels ranging from 1.0 to 6.3 µg/kg and an average concentration of 5.3 µg/kg. Three barley couscous samples (60%) also tested positive for TEN, with concentrations between 1.3 and 1.9 µg/kg (mean: 1 µg/kg). No contamination with TEN was detected in the corn couscous samples.

### 2.5. Co-Occurrence of Mycotoxins in Couscous Samples

Regarding multi-mycotoxin contamination in couscous samples, only one sample (industrial wheat couscous) was not contaminated with mycotoxins, while only one mycotoxin was detected in four samples (one hand-rolled, two industrial, and one barley couscous). The highest incidences were recorded for TEN and DON (76 and 74%, respectively), followed by TeA, OTA, HT2, AFB_1_, T2, AFB_2_, FB_1_, AFG_1_, and AFG_2_.

In the hand-rolled couscous samples, all the samples were contaminated with at least three mycotoxins. The most frequently observed combination was TeA, TEN, and DON. Additionally, co-contamination with five mycotoxins was detected in two samples: one containing TeA, TEN, DON, OTA, and AFB_1_; and another with TeA, TEN, DON, AFB_1_, and AFB_2_.

All industrial wheat couscous samples (*n* = 23) were contaminated with at least one mycotoxin. The most frequently observed mycotoxin combination was (TeA, TEN, DON), followed by the combination (TeA, TEN, HT-2, DON, OTA). One sample (4.3%) contained seven different mycotoxins (TeA, TEN, HT-2, T-2, DON, OTA, AFB_1_), while another sample (4.3%) contained six mycotoxins (TeA, TEN, HT-2, T-2, DON, OTA). Three samples (13.0%) were contaminated with five mycotoxins, one sample (4.3%) with four and five samples (21.7%) with three mycotoxins. In addition, two samples (8.7%) contained two mycotoxins, and three samples (13.0%) were contaminated with only a single mycotoxin.

All the analyzed organic wheat couscous samples (*n* = 3) were simultaneously contaminated with five different mycotoxins (TeA, TEN, HT-2, DON, OTA).

Among the analyzed corn couscous samples, two samples (50%) were co-contaminated with six different mycotoxins. The combinations identified were: (FB_1_, FB_2_, DON, AFB_1_, AFB_2_, AFG_1_) and (FB_1_, FB_2_, AFB_1_, AFB_2_, AFG_1_, AFG_2_). One sample (25%) was contaminated with three distinct mycotoxins, while another (25%) contained two different mycotoxins.

For the barley couscous, two samples (40%) were co-contaminated with the following combinations of five different mycotoxins: (TEN, HT-2, T2, DON, OTA) and (TEN, TeA, HT-2, DON, OTA). Two additional samples (40%) were contaminated with three distinct mycotoxins, while one sample (20%) contained only a single mycotoxin.

All whole wheat couscous samples were contaminated with at least three different mycotoxins. One sample (25%) contained six mycotoxins (TeA, TEN, HT-2, DON, OTA, AFB1), while another (25%) was contaminated with five mycotoxins (TeA, TEN, HT-2, DON, OTA). Additionally, one sample was contaminated with four mycotoxins, and the remaining sample contained three mycotoxins.

Pearson’s correlation underlined a correlation among conventional/organic farming and the contents of TEN and DON (*p* < 0.05), while HT2 was correlated with the main ingredient used for couscous preparation. No correlation was found between the production method of couscous (industrial/hand rolled) or the size of couscous grains and the different mycotoxins (Table S1).

## 3. Discussion

Mycotoxins are among the major biological contaminants affecting the safety and quality of cereal-based products [18]. Given their well-documented adverse health effects, the present study aimed to assess the mycotoxin contamination patterns in this commonly consumed cereal-based food. Although widely consumed in North Africa, couscous has received limited scientific attention concerning mycotoxin contamination, unlike raw cereals which have been more extensively studied.

In line with these objectives, a detailed assessment of contamination profiles was conducted on various types of couscous marketed in Algeria. Among the targeted mycotoxins, no traces of nivalenol (NIV), alternariol (AOH), or alternariol monomethyl ether (AME) were detected, suggesting an absence of contamination by these compounds across all analyzed matrices.

Based on the analytical results, all hand-rolled wheat couscous samples (100%, *n* = 11) were contaminated with mycotoxins produced by various fungal genera. The most frequently detected mycotoxins were tenuazonic acid (TeA), present in 81.8% of the samples, and deoxynivalenol (DON), found in 63.6%, which were attributed to *Alternaria* and *Fusarium* species, respectively. Regarding the TeA levels, the mean concentration measured in the hand-rolled traditional wheat couscous samples (71.8 ± 59.0 µg/kg) was lower than that reported by Daichi et al. [19], who analyzed 48 durum wheat samples from various Algerian regions and recorded an average level of 177 µg/kg. Similarly, the same authors also found a mean tentoxin (TEN) concentration of 38 µg/kg, which is much higher than the mean concentration observed in the present study (2.9 ± 1.5 µg/kg). To date, no regulatory limits have been established for *Alternaria* mycotoxins in foodstuffs intended for human or animal consumption. However, the European Union, through Commission Recommendation (EU) 2022/553, requests monitoring for AOH, AME, and TeA in specific food products and reports indicative levels in certain specific food products [20].

None of the DON concentrations measured in the present study exceeded the maximum limit of 600 µg/kg established by Regulation (EU) 2024/1022 [21]. The mean concentration observed in the hand-rolled traditional wheat couscous samples (47.7 ± 59.0 µg/kg) was higher than that reported in wheat couscous samples collected in Morocco (14.9 µg/kg) [8], but lower than the level recorded by Belasli et al. [2] in similar samples collected in Algeria (117 ± 97 µg/kg). Moreover, the levels detected in this study are well below those reported by Khouni et al. [10], who observed a concentration of 1076 µg/kg in a wheat sample stored in 2020 in the Mediterranean region of Algeria.

The results of the present study indicate that among the traditional couscous samples analyzed, two (18.2%) were contaminated with aflatoxin B_1_ (AFB_1_), with a mean concentration of 0.08 ± 0.16 µg/kg, which is below the maximum regulatory limit of 2 µg/kg set by Regulation (EU) 2023/915 [22]. In addition, aflatoxin B_2_ (AFB_2_) was detected in a single sample (9.1%), also below the regulatory thresholds established by the European Union. These findings contrast with those reported by Belasli et al. [2] and Zinedine et al. [8], who did not detect any traces of AFB_1_ or AFB_2_ in wheat couscous samples collected in Algeria (*n* = 27) and Morocco (*n* = 84), respectively. In contrast, Khouni et al. [10] reported higher levels, with a mean AFB_1_ concentration of 1.2 µg/kg detected in a post-harvest wheat sample (*n* = 20) collected in 2021 in the continental region of Algeria.

HT-2 and T-2 toxins were detected in only one sample (9.1%) at very low concentrations, well below the regulatory limit of 20 µg/kg set for the sum of both compounds, in accordance with Regulation (EU) 2024/1038 [23]. These findings are lower than those reported by Mahdjoubi et al. [5], who found that 23% of wheat samples (*n* = 30) were contaminated with HT-2 toxin at concentrations ranging from 8.4 to 36.7 µg/kg, while T-2 toxin was detected in all samples (100%) at levels ranging from 16.6 to 47.5 µg/kg.

Ochratoxin A (OTA) was detected in 54.5% of the samples analyzed, with concentrations consistently below the maximum limit of 3 µg/kg established by Regulation (EU) 2023/915 [22]. Similar results were reported by Belasli et al. [2], who observed a mean concentration of 0.20 ± 0.56 µg/kg in wheat couscous samples collected in Algeria. In contrast, Mahdjoubi et al. [5] did not detect any OTA contamination in wheat samples collected from the same country. Higher concentrations than those found in the present study were reported by Khouni et al. [10], who detected OTA in 9.9% of wheat samples, including one post-harvest sample with a concentration of 3.8 µg/kg, exceeding the regulatory limit set by the European Union. Additionally, a single sample of unprocessed wheat collected in Tunisia contained OTA at a level well below the maximum threshold authorized by European legislation [24].

A similar contamination pattern was observed in industrial wheat couscous (Table 2); however, the mean levels of TeA and DON were lower (39.1 ± 27.0 and 35.9 ± 42.6 µg/kg, respectively) than those detected in hand-rolled couscous. Furthermore, DON contamination levels are below the maximum limits established by European regulations [21].

Although organic farming is increasingly valued for reducing the use of chemical products [25], no comparative study on mycotoxin contamination in organic versus conventional products has yet been conducted in Algeria. In this context, the analysis of organic wheat couscous samples (Table 3) revealed consistent contamination with DON, TeA, TEN, HT-2, and OTA. This is partially in agreement with a previous study conducted in Italy on maize that showed a significantly higher contamination in organic farming samples than in conventional ones for fumonisins [26]. The mean levels of DON (200.3 µg/kg) and TeA (117.5 µg/kg) were higher than those found in the hand-rolled and industrial couscous (Figure 1), although the DON concentrations are below the regulatory limit established by the European Union [21]. These values are comparable with those reported by Pleadin et al. [27] in unprocessed organic wheat (256 ± 204 µg/kg), but higher than those found in organic wheat flour (68.9 ± 46.4 µg/kg).

All four corn couscous samples analyzed (*n* = 4) were contaminated with at least two different mycotoxins. Fumonisins (FB_1_ and FB_2_) were detected in all the samples, with concentrations below the maximum permissible level of 1000 µg/kg for maize-based products, as established by Regulation (EU) 2023/915 [22] (Table 4). These levels are comparable to those reported in Morocco, where mean concentrations of 464.7 µg/kg for FB_1_ and 168.1 µg/kg for FB_2_ were observed in maize couscous samples [8]. In contrast, our findings are lower than those reported by Mahdjoubi et al. [5], who analyzed 30 unprocessed maize samples from Algeria and reported mean levels of 14,812 µg/kg for FB_1_ and 2789 µg/kg for FB_2_. In that study, 70% of samples exceeded the European maximum limit of 4000 µg/kg set for unprocessed maize [22].

Conversely, the FB levels detected in our study were higher than those found in Croatia, where a mean concentration of 168 ± 114 µg/kg (FB_1_ + FB_2_) was reported in conventional maize semolina [27]. Regarding aflatoxins, AFB_1_, AFB_2_, and AFG_1_ were detected in 50% of the maize couscous samples, with concentrations consistently below the EU regulatory limit of 2 µg/kg [24]. Specifically, the AFB_1_ levels in our samples were lower than those reported in Morocco (mean: 5.2 µg/kg) [8] and in the study by Jedidi et al. [26], where a mean of 4.65 ± 4.65 µg/kg was found in unprocessed maize. The latter study suggested that recommended seed treatments applied prior to storage or retail distribution could help to improve the microbial safety of maize products intended for human consumption. The detection of AFB_1_ in maize-based couscous reflects the well-known vulnerability of maize to *Aspergillus flavus* contamination. This aligns with global data highlighting the persistent risk of aflatoxin exposure due to the widespread presence of toxigenic fungi in maize production and storage environments [28,29].

Finally, DON was detected in two samples at very low concentrations (mean: 9 µg/kg), markedly lower than those measured in wheat-based products. However, these levels remain higher than those reported by Zinedine et al. [8] who observed no DON contamination in the maize couscous samples they analyzed. In contrast, the study by Pleadin et al. [27] reported a considerably higher mean concentration of 80.3 ± 83.9 µg/kg.

No aflatoxins were detected in the barley samples (*n* = 5), all of which originated from locally grown crops and were traditionally processed. These findings are consistent with those reported in Tunisia, where no aflatoxins were detected in barley samples [24], but contrast with the results obtained in Morocco, where Zinedine et al. [8] reported concentrations exceeding the regulatory limit of 4 µg/kg in 3 out of 8 barley couscous samples. Although their occurrence appears to be sporadic, the detection of aflatoxins in wheat and barley underscores the need for rigorous and ongoing monitoring of these contaminants in cereal matrices [24]. In the present study, ochratoxin A (0.24 µg/kg), HT-2 toxin (4.36 µg/kg), and T-2 toxin (1.03 µg/kg) were detected at trace levels, remaining below regulatory thresholds [22]. However, these values remain higher than those reported in Moroccan barley couscous, where no OTA, HT-2, or T-2 contamination was observed [8]. Similarly, Mahdjoubi et al. [5] did not detect any of these mycotoxins in barley samples collected in western Algeria.

All whole-wheat couscous samples exhibited co-occurrence of mycotoxins, with a minimum of three distinct types detected. The highest mean concentrations were observed for TeA, at 47.8 µg/kg, followed by DON, at 38.9 µg/kg (Table 6). These levels are comparable to those reported in wheat-based couscous, whether traditionally handmade or industrially processed (Figure 1). Contrary to our findings, several studies have shown that whole-grain flours generally exhibit higher mycotoxin levels than refined (white) flours, due to the greater accumulation of contaminants in the outer layers of the grain. Although certain milling practices can partially reduce the mycotoxin content, they are not sufficient to achieve complete elimination [30]. Nevertheless, the concentration observed in this study is lower than those found in couscous prepared from organic wheat or maize. Conversely, they are higher than those reported for barley-based couscous.

It is important to emphasize that the synthesis of previously cited studies highlights that unprocessed cereals consistently exhibit higher mycotoxin levels than processed products. This reduction is largely attributed to the effectiveness of physical removal methods, such as manual or automated sorting, as well as processing steps including milling, soaking, and extrusion, which collectively reduce mycotoxin concentrations [31]. In particular, the low levels of some mycotoxins, such as TeA, TEN, DON, FBs, and OTA, found in couscous samples could be explained by their solubility in water and consequent loss during the preparation step of wet agglomeration, during which semolina is mixed with water to form moist agglomerates.

Co-contamination with multiple mycotoxins was consistently observed across all the couscous samples analyzed, regardless of the cereal type or production method (artisanal, industrial, organic, or whole grain). The most frequently detected combination included TeA, TEN, and DON.

The co-occurrence of mycotoxins in cereals has been extensively documented, particularly in Mediterranean regions. In a recent study, Khouni et al. [10] reported that 50% of wheat samples (*n* = 30), collected post-harvest in a Mediterranean area of Algeria during the 2020 season were contaminated with at least one mycotoxin. Additionally, 10% of the samples exhibited co-contamination with seven different mycotoxins. Consistent with our findings, a study conducted in Italy showed that 81% of cereal samples were contaminated with more than one mycotoxin [27]. Several studies demonstrated that the co-occurrence of multiple mycotoxins may result in toxic effects that are not only additive but also synergistic, whereby the overall toxicity may exceed the expected additive outcome of individual effects and lead to an amplified biological response [32,33,34].

Despite the relatively small number of samples for certain couscous types, this study underscores the need for a comprehensive and regular national surveillance program to monitor mycotoxin contamination in couscous marketed in Algeria in order to safeguard food safety and public health. Furthermore, it provides preliminary insights into the occurrence of mycotoxins in a major cereal-based staple. These limitations highlight the importance of expanding future studies to include a broader range of regions and couscous types, thereby generating more representative data.

## 4. Conclusions

This is one of the first studies to investigate the co-occurrence of 15 mycotoxins—including commonly monitored mycotoxins (AFB_1_, AFB_2_, AFG_1_, AFG_2_, OTA, DON, NIV, HT-2, T-2, FB_1_, FB_2_) and emerging *Alternaria* toxins (TeA, TEN, AOH, AME)—in couscous, a traditional cereal-based food widely consumed in Algeria. While all detected levels remained below current EU regulatory limits, co-contamination was widespread: 90% of the samples contained two or more mycotoxins, with some exhibiting up to seven. These results raise concerns about the potential cumulative dietary exposure and associated health risks.

To mitigate these risks, greater awareness among stakeholders throughout the food chain and implementing good agricultural and storage practices are essential. Algerian authorities are also encouraged to define national maximum limits for these contaminants in food and feed. Further research involving larger sample sets from various regions to ensure more representative data, along with dietary exposure assessments, is recommended to better characterize the risks and support evidence-based decision-making in food safety.

## 5. Materials and Methods

### 5.1. Sampling

A total of 50 couscous samples were randomly collected in 2024 from various supermarkets and retail outlets across nine administrative regions in Algeria: Algiers, Boumerdès, Blida, Bouira, Tizi Ouzou, Sétif, Constantine, Oran, and Médéa. Sampling was conducted in northern Algerian regions, covering both Mediterranean and continental climates. These regions are major cereal-producing areas, densely populated, and central to national food consumption. They also host well-developed couscous and pasta industries, including key centers such as Algiers, Blida, Oran, Boumerdès, and Tizi Ouzou. Together, these factors ensure the selected samples are representative of the national couscous market. The production process involves several sequential steps. The first step is wet agglomeration, during which semolina is mixed with water to form moist agglomerates. These agglomerates are then rolled to ensure a homogeneous grain structure [35,36]. The second step consists of a hydrothermal treatment at 180 °C for 8 min. This process enables partial precooking of the product [16]. Finally, a drying step is carried out to reduce the moisture content to approximately 13%. The dried couscous is then subjected to sieving, allowing classification into different grain sizes (fine, medium, or coarse) [37]. In this study, the sampling included different types of couscous, namely, hand-rolled wheat couscous (*n* = 11); industrial wheat couscous (*n* = 23; fine, medium, and large grain); organic couscous (*n* = 3); and couscous made from corn (*n* = 4), barley (*n* = 5), and whole wheat (*n* = 4). The numbers of samples for each category were based on their market uptake and consumption by the local population. According to field information provided by vendors and manufacturers, wheat used in the couscous production was either locally grown or imported; the barley originated exclusively from local cultivation, whereas corn was imported. All the samples were stored in polyethylene bags and maintained at 4 °C until analysis.

The samples were analyzed for the presence of the following mycotoxins: aflatoxins (AFs), ochratoxin A (OTA), deoxynivalenol (DON), nivalenol (NIV), HT-2 and T-2 toxins, fumonisins (FBs), and *Alternaria* toxins (ALTs; tenuazonic acid (TeA), tentoxin (TEN), alternariol (AOH), alternariol monomethyl ether (AME)).

Samples were milled using a cyclone hammer mill equipped with a 1 mm sieve, homogenized, and stored at −20 °C until analysis.

### 5.2. Analysis for AFs, FBs and OTA Determination

Mycotoxins were quantified using previously validated methods developed in our laboratory [38,39]. Briefly, AFBs were extracted from 25 g sample with 250 mL water–acetone (analysis grade) 7 + 3 *v*/*v* under agitation for 1 h, then purified in an immunoaffinity (IA) column (Easi-Aflatoxin, R-Biopharm, Rhône Ltd., Glasgow, Scotland, UK) and eluted in a graduated glass vial using 3 mL methanol. Finally, AFs were analyzed using HPLC-FLD (Jasco Corporation, Tokyo, Japan) with photochemical derivatization. FBs (FB_1_ and FB_2_) and OTA were extracted from the 20 g sample with phosphate buffer pH 7.4 (200 mL) and 0.13 M sodium bicarbonate–methanol (analysis grade) 5 + 5 *v*/*v* (200 mL), respectively, with both purified in an IA column (R-Biopharm, Darmstadt, Germany). FBs were separated in a RP-18 column and quantified using LC-MS/MS (Thermo Fisher Scientific, San Jose, CA, USA) (positive mode), while OTA was chromatographed in a RP-18 column using a mobile phase acetonitrile–acidified water (2% acetic acid) in gradient mode and quantified using HPLC-FLD.

The LOD and LOQ were, respectively, 0.05 and 0.10 µg/kg for AFs, 2.0 and 5.0 µg/kg for FBs, and 0.02 and 0.04 µg/kg for OTA. Calibration curves showed an r value > 0.998. Because of the IA purification step, the mean recovery values were > 95.5 ± 3.8% and no matrix effect was observed.

### 5.3. Analysis for ALTs Determination

ALTs were extracted from 25 g of milled sample with 100 mL acetonitrile: water 80 + 20 *v*/*v* using a rotary shaker for 60 min (120 rpm), as reported by [40]. The extract was centrifuged at 4000 rpm, then 0.200 mL was diluted with 0.600 mL water: acetonitrile 90:10 *v*/*v* in vial for LC-MS/MS analysis (Vanquish pump and autosampler coupled to Fortis mass spectrometer, Thermo Fisher Scientific, San Jose, CA, USA) in SRM (selected reaction monitoring) mode. *Alternaria* toxins were chromatographed on an HSS-T3 RP-18 column (5 µm particle size, 150 × 2.1 mm, Waters Corp, MA, USA) using an acetonitrile-water mobile phase gradient (both acidified with 0.2% formic acid) from 30:70 to 65:35 in 6 min, then isocratic for 3 min; gradient to 30:70 in 1 min and isocratic for 6 min (equilibrium phase). Ionization was carried out with an ESI interface (Thermo-Fisher, San Jose, CA, USA) in positive mode as follows: spray capillary voltage 4.5 kV, sheath and auxiliary gas 35 and 14 psi, respectively, heated capillary temperature 270 °C. For fragmentation of [M + H]^+^ ions (198 *m*/*z* for TeA, 259 *m*/*z* for AOH, 273 *m*/*z* for AME, and 415 *m*/*z* for TEN), fragment ions were: 125, 139 and 153 *m*/*z* (16 V) for TeA, 128, 185 and 213 *m*/*z* (35 V) for AOH, 128, 184 *m*/*z* (38 V) and 258 *m*/*z* (30 V) for AME, 132 *m*/*z* (37 V), 135 and 312 *m*/*z* (25 V) for TEN. Quantitative determination was performed by LC_Quan 2.0 software.

To study the matrix effect (ME), the slopes of matrix-matched calibration curves obtained from 5 spiked blank sample extracts (3 replicates for each sample) were compared with those of solvent-based calibration curves by calculating ME with the following formula: ME (%) = (matrix standard curves slope/solvent standard curves slope) × 100%. The results showed that ME was always less than 7%; then, the results were considered unaffected by the matrix.

The limit of detection (LOD) and limit of quantification (LOQ) were determined by the signal-to-noise approach, defined as those levels that result in signal-to-noise ratios of 3 and 10, respectively. Chromatographic noise and analytical response were analyzed from the chromatogram of a blank sample extract to which an appropriate volume of alternariol standard solution had been added. The LOD and LOQ were 5 and 15 µg/kg for TeA, 0.5 µg/kg and 1.5 µg/kg for AOH and TEN, 1 and 2.5 µg/kg for AME. The linearity of the calibration curves was established through five solvent calibration standards, showing r values greater than 0.998.

The concentration levels were: 25, 50, 100, 250 and 500 µg/l for TeA, 1, 2.5, 5, 10 and 25 µg/L for AOH, AME and TEN. Recovery values were determined by spiking the aliquot of an uncontaminated (blank) sample (20 g) with an appropriate volume of alternariol standard solution at three different levels before the extraction; three replicates were analysed for each level. The recovery values were between 95.4–99.7%. The results were not corrected for the mean recovery value.

### 5.4. Analysis for DON, NIV, HT-2 and T-2 Determination

As for ALTs, TCTs were extracted from 25 g of ground sample with 100 mL of acetonitrile: water 80 + 20 *v*/*v*. Filtration on filter paper was then performed, and the extract (0.200 mL) was diluted with methanol: water 10 + 90 *v*/*v* mixture (0.400 mL) before LC-MS/MS determination (Vanquish pump and autosampler coupled to Fortis mass spectrometer, Thermo Fisher Scientific, San Jose, CA, USA). Chromatographic separation was carried out using a Betasil RP-18 column (5 µm particle size, 150 × 2.1 mm, Thermo-Fisher) with a 10 mM methanol-ammonium acetate mobile phase gradient (pH 6.8) from 10:90 (isocratic 2 min) to 65:35 in 4 min, then isocratic for 3 min, gradient to 10:90 in 1 min and isocratic for 6 min (conditioning phase). Ionization was carried out with an H-ESI interface (Thermo-Fisher) in negative mode as follows: spray capillary voltage 3.1 kV, sheath and auxiliary gas 35 and 15 psi, respectively, vaporizer temperature 200 °C and ion transfer tube temperature 270 °C. For DON, fragmentation ions were: 247, 265 and 295 *m*/*z* (parent ion 355 *m*/*z*, acetate adducted), collision gas (argon) was 1.5 psi and collision energy were 12 to 16 V. For NIV, fragmentation ions were: 281 and 311 *m*/*z* (parent ion 371 *m*/*z*, acetate adducted). The calibration levels were: 10, 25, 50, 100 and 250 µg/l for both DON and NIV. The recovery values were between 90.1–97.7%. Regarding HT-2 and T-2, the quantification was carried out applying the method of [41] in positive mode. As for ALTs, matrix effect was not considered, because inferior to 5%. The LOD was 5 µg/kg for DON and NIV, 0.1 µg/kg for HT-2 and T-2.

### 5.5. Statistical Analysis of Data

The data on mycotoxins production was logarithmically transformed prior to the statistical analysis (values +1) [42]. Because of the low number of samples and the non-normal distribution of the results, analysis of variance (ANOVA) was not performed. Pearson correlations between all the main factors considered in the study (ingredients, type of cultivation, and method of preparation) and the different mycotoxins were calculated. The correlations were considered significant at the 0.05 level. All data were analysed using the IBM SPSS Statistics 27 package (IBM Corp., Armonk, NY, USA).

## Figures and Tables

**Table 1:** Mycotoxin contamination (µg/kg) in hand rolled wheat couscous.

Hand Rolled Wheat (*n* = 11)	TeA	TEN	HT2	T2	DON	OTA	AFB_1_	AFB_2_
Hand rolled couscous + lavender	<5	1.1	<0.1	<0.1	<5	<0.02	<0.05	<0.05
Hand rolled couscous	<5	2.4	8.65	2.67	194.1	0.68	<0.05	<0.05
Hand rolled couscous + basil	80.6	4.2	<0.1	<0.1	46.8	<0.02	<0.05	<0.05
Hand rolled couscous + oregano	24.8	1.8	<0.1	<0.1	30.4	<0.02	<0.05	<0.05
Hand rolled couscous + basil	63.4	4.8	<0.1	<0.1	92.7	0.09	0.11	<0.05
Hand rolled couscous + thyme	26.8	2.2	<0.1	<0.1	35.6	0.24	<0.05	<0.05
Hand rolled couscous	148.1	4.3	<0.1	<0.1	93.1	<0.02	0.56	0.10
Hand rolled couscous	152.9	4.7	<0.1	<0.1	<5	0.09	<0.05	<0.05
Hand rolled couscous	162.7	4.2	<0.1	<0.1	<5	0.33	<0.05	<0.05
Hand rolled couscous	65.8	1.2	<0.1	<0.1	<5	0.06	<0.05	<0.05
Hand rolled couscous	59.5	1.0	<0.1	<0.1	22.4	<0.02	<0.05	<0.05
% Positives	81.81	100	9.09	9.09	63.63	54.54	9.09	9.09
Mean ± std dev *	71.8 ± 59.0	2.9 ± 1.5	0.83 ± 2.59	0.29 ± 0.79	47.7 ± 59.0	0.14 ± 0.21	0.08 ± 0.16	0.03 ± 0.02

* For samples contaminated < LOD, the mean and std dev were calculated using the middle bound approach. Tenuazonic acid (TeA); tentoxin (TEN); deoxynivalenol (DON); ochratoxin A (OTA); alfatoxin B_1_ (AFB_1_); aflatoxin B_2_ (AFB_2_).

**Table 2:** Mycotoxin contamination (µg/kg) in industrial wheat couscous.

	TeA	TEN	HT2	T2	DON	OTA	AFB_1_
Fine couscous (*n* = 4)	15.8	1.9	<0.1	<0.1	8.3	<0.02	<0.05
	21.0	<0.5	<0.1	<0.1	8.4	<0.02	<0.05
	69.3	2.0	<0.1	<0.1	20.4	<0.02	<0.05
	55.9	1.6	<0.1	<0.1	<5	<0.02	<0.05
% Positives	100	100	0	0	25	0	0
Mean ± std dev	40.5	1.5	0.05	0.05	9.9	0.01	0.025
Medium couscous (*n* = 16)	43.2	6.3	1.52	0.40	75.3	0.28	0.16
	34.0	2.7	<0.1	<0.1	17.2	<0.02	0.12
	51.4	2.4	0.96	0.10	37.6	0.22	<0.05
	<5	<0.5	<0.1	<0.1	<5	<0.02	<0.05
	<5	<0.5	<0.1	<0.1	69.7	0.14	<0.05
	24.5	1.8	<0.1	<0.1	<5	<0.02	<0.05
	48.5	1.6	<0.1	<0.1	63.3	<0.02	<0.05
	49.6	1.0	<0.1	<0.1	39.4	<0.02	<0.05
	48.6	1.0	<0.1	<0.1	94.3	<0.02	<0.05
	<5	<0.5	<0.1	<0.1	6.3	<0.02	<0.05
	<5	<0.5	<0.1	<0.1	8.0	<0.02	<0.05
	117.4	3.2	1.84	0.28	192.4	0.37	0.05
	21.8	1.1	0.85	0.80	34.6	0.19	<0.05
	45.5	2.2	<0.1	<0.1	37.7	0.04	<0.05
	57.7	1.5	0.46	<0.1	19.6	0.21	<0.05
	63.6	1.2	0.30	<0.1	24.8	0.03	<0.05
% Positives	75	75	37.5	25	87.5	50	18.8
Mean ± std dev	38.5	1.7	0.4	0.1	45.3	0.10	0.040
Large couscous (*n* = 3)	22.1	<0.5	<0.1	<0.1	5.0	0.06	0.15
	48.2	1.0	<0.1	<0.1	33.6	<0.02	<0.05
	50.6	1.5	<0.1	<0.1	22.2	<0.02	<0.05
% Positives	100	66.7	0	0	100	33.3	33.3
Mean ± std dev *	40.3	1,0	0.05	0.05	20.3	0.03	0.066
Global Mean ± std dev	39.1 ± 27.0	1.6 ± 1.3	0.30 ± 0.51	0.11 ± 0.17	35.9 ± 42.6	0.07 ± 0.11	0.041 ± 0.040

* For samples contaminated < LOD, the mean and std dev were calculated using the middle bound approach.

**Table 3:** Mycotoxin contamination (µg/kg) in organic wheat couscous.

	TeA	TEN	HT2	T2	DON	OTA
Couscous with organic wheat (*n* = 3)	78.9	6.0	1.36	<0.1	65.7	0.10
	141.5	4.3	2.01	<0.1	281.5	0.21
	132.0	5.7	1.60	<0.1	253.7	0.36
% Positives	100	100	100	0	100	100
Mean ± std dev *	117.5	5.3	1.66	0.05	200.3	0.22

* For samples contaminated < LOD, the mean and std dev were calculated using the middle bound approach.

**Table 4:** Mycotoxin contamination in corn couscous.

	FB1	FB2	DON	AFB1	AFB2	AFG1	AFG2
Corn + rice	37.4	8.7	10.9	<0.05	<0.05	<0.05	<0.05
Hand rolled couscous with corn	474.8	196.8	20.0	1.13	0.09	0.40	<0.05
Hand rolled couscous with corn	659.9	249.9	<5	0.84	0.05	0.45	<0.05
Corn + rice	67.3	26.7	<5	<0.05	<0.05	<0.05	<0.05
% Positives	100	100	50	50	50	50	25
Mean ± std dev *	309.8	120.6	9.0	0.51	0.05	0.22	0.03

* For samples contaminated < LOD, the mean and std dev were calculated using the middle bound approach.

**Table 5:** Mycotoxin contamination in barley couscous.

	TeA	TEN	HT2	T2	DON	OTA
Hand rolled couscous with barley (*n* = 5)	<5	1.3	8.44	2.77	28.6	0.28
	19.3	1.6	1.44	<0.1	60.1	0.25
	<5	1.4	<0.1	<0.1	<5	<0.02
	<5	<0.5	4.63	0.58	<5	0.32
	<5	<0.5	7.22	1.71	<5	0.36
% Positive	20	60	80	60	40	80
Mean ± std dev *	5.9	1.0	4.36	1.03	19.2	0.24

* For samples contaminated < LOD, the mean and std dev were calculated using the middle bound approach.

**Table 6:** Mycotoxin contamination in whole wheat.

	TeA	TEN	HT2	T2	DON	OTA	AFB1
Couscous with whole wheat (*n* = 4)	44.8	6.6	1.93	<0.1	88.6	0.26	0.09
	41.2	2.3	<0.1	<0.1	12.2	0.07	<0.05
	54.3	5.0	1.29	<0.1	52.2	0.16	<0.05
	50.8	2.4	<0.1	<0.1	<5	0.28	<0.05
% Positive	100	100	50	0	25	100	25
Mean ± std dev *	47.8	4.1	0.83	0.05	38.9	0.19	0.04

* For samples contaminated < LOD, the mean and std dev were calculated using the middle bound approach.

## Data Availability

The original contributions presented in this study are included in the article. Further inquiries can be directed to the corresponding authors.

## Supplementary Materials

The following supporting information can be downloaded at: https://www.mdpi.com/article/10.3390/toxins17100483/s1, Table S1: Analysis of Pearson’s correlation among the main characteristics of couscous products and mycotoxins contamination.
