# Supplementary material for: Co-Occurrence of Major Mycotoxins and Emerging Alternaria Toxins in Couscous Marketed in Algeria
Source: Toxins (Basel). 2025 Sep 26;17(10):483. doi: 10.3390/toxins17100483 (PMC12568311; doi:10.3390/toxins17100483)
Supplement: Supplementary file 1 [file toxins-17-00483-s001.zip › toxins-3857830-supplementary.pdf]

toxins

MDPI

Co-Occurrence of Major Mycotoxins and Emerging *Alternaria* Toxins in Couscous Marketed in Algeria

Sarah Mohammedi-Ameur [1,2,*], Terenzio Bertuzzi [3,*], Roberta Battaglia [3], Federico Siboni [3], Paola Giorni [4] and Dahmane Mohammedi [2]

[1] École Supérieure des Sciences de l'Aliment et des Industries Agroalimentaires (ESSAIA, Algiers), 16200, Oued Smar, Algiers,Algeria
[2] Laboratoire de Recherche Santé et Productions Animales, École Nationale Supérieure Vétérinaire Rabie Bouchama (ENSV, Algiers),16042 Bab Ezzouar, Algiers,Algeria; mohammedidahmane@yahoo.fr
[3] Department of Animal Science, Food and Nutrition (DIANA), Faculty of Agriculture, Food and Environmental Sciences, Università Cattolica del Sacro Cuore, 29122 Piacenza, Italy
[4] Department of Sustainable Crop Production (DI.PRO.VE.S.), Faculty of Agriculture, Food and Environmental Sciences, Università Cattolica del Sacro Cuore, 29122 Piacenza, Italy; paola.giorni@unicatt.it
* Correspondence: mohammedi@essaia.dz (S.M.-A.); terenzio.bertuzzi@unicatt.it (T.B.)

Table S1 – Analysis of Pearson's correlation among the main characteristics of couscous products and mycotoxins contamination.

		**TeA**	**TEN**	**HT2**	**T2**	**DON**	**OTA**	**AFB1**
Main ingredient (Barley, Whole wheat, Corn-Rice, Durum wheat)	R	-0,243	0,001	0,341*	0,190	-0,187	0,268	0,049
	p	0,090	0,996	0,015	0,186	0,193	0,060	0,735
Kind of cultivation (Conventional/organic)	R	0,258	0,364**	0,247	-0,089	0,342*	0,200	-0,084
	P	0,071	0,009	0,084	0,539	0,015	0,154	0,564
Type of preparation (industrial/hand rolled)	R	0,187	0,300*	-0,130	0,020	-0,031	0,060	0,003
	p	0,193	0,034	0,370	0,892	0,832	0,678	0,983

R: Pearson's coefficient, p: test probability at a significance level of $\alpha = 0.05$ and * significant ($p < 0.05$).
